# Impact of Catheter Ablation on Functional Capacity and Cardiac Stress Markers in Patients with Premature Ventricular Contractions

**DOI:** 10.3390/medsci13030095

**Published:** 2025-07-23

**Authors:** Vasileios Cheilas, Athanasios Dritsas, Antonios Martinos, Evangelia Gkirgkinoudi, Giorgos Filandrianos, Anastasios Chatziantoniou, Ourania Kariki, Panagiotis Mililis, Athanasios Saplaouras, Anna Kostopoulou, Konstantinos Letsas, Michalis Efremidis

**Affiliations:** 1 Onassis Cardiac Surgery Center, 17674 Athens, Greece; 2 Artificial Intelligence Lab, National Technical University of Athens, 15780 Athens, Greece

**Keywords:** catheter ablation, premature ventricular contractions (PVCs), cardiopulmonary exercise testing (CPET), functional capacity, NT-proBNP

## Abstract

**Background:** Premature ventricular contractions (PVCs) are common arrhythmias associated with symptoms such as fatigue and, in severe cases, PVC-induced cardiomyopathy. Catheter ablation (CA) is a primary treatment for symptomatic PVCs, particularly when pharmacological therapies fail or are undesired. While improvements in: quality-of-life following ablation are documented, its impact on functional capacity remains underexplored. **Objectives:** This study evaluated the impact of CA on functional capacity and cardiac stress markers in patients with symptomatic PVCs using cardiopulmonary exercise testing (CPET) and NT-proBNP levels. **Methods:** A total of 30 patients underwent successful PVC ablation and completed baseline and follow-up CPET evaluations under the Bruce protocol. PVC burden, left ventricular ejection fraction (LVEF), NT-proBNP levels, and CPET parameters, including VO_2_ max, METS, ventilatory efficiency, and anaerobic threshold (AT), were analyzed pre- and post-ablation. **Results:** PVC burden significantly decreased post-ablation (23,509.3 ± 10,700.47 to 1759 ± 1659.15, *p* < 0.001). CPET revealed improved functional capacity, with VO_2_ max increasing from 24.97 ± 4.16 mL/kg/min to 26.02 ± 4.34 mL/kg/min (*p* = 0.0096) and METS from 7.16 ± 1.17 to 7.48 ± 1.24 (*p* = 0.0103). NT-proBNP significantly decreased (240.93 ± 156.54 pg/mL to 138.47 ± 152.91 pg/mL, *p* = 0.0065). LVEF and ventilatory efficiency metrics (VE/VO_2_ and VE/VCO_2_) remained stable. **Conclusions:** Catheter ablation improves functional capacity, reduces cardiac stress, and minimizes medication dependency in patients with symptomatic PVCs. These findings support the utility of ablation in enhancing aerobic capacity and overall exercise performance.

## 1. Introduction

Premature ventricular contractions (PVCs) are among the most frequently encountered arrhythmias in clinical practice, associated with a range of symptoms, from palpitations to fatigue, and, in severe cases, PVC-induced cardiomyopathy [1]. Catheter ablation (CA) has emerged as a primary treatment for symptomatic PVCs [2,3]. In addition to reducing PVC burden, CA has been shown to restore left ventricular (LV) function in patients with PVC-induced cardiomyopathy [4] and reduce episodes of PVC-related ventricular fibrillation [5].

The success rates of PVC ablation range between 75 and 92%, with low procedural complication rates [6]. However, outcomes depend on the origin of the PVC, with the highest success observed in outflow tract PVCs, while epicardial and papillary muscle PVCs come with smaller success rates [6]. Although improvements in LV function following CA have been documented, data on the effects of ablation on functional capacity, particularly exercise tolerance, are sparse and mostly limited to case reports [7].

This study aims to address this gap by evaluating the impact of CA on functional capacity through cardiopulmonary exercise testing (CPET). To the best of our knowledge, although small, this represents the first real-world, single-center study to assess these outcomes.

## 2. Methods

### 2.1. Study Population

This prospective, single-center study included *n* = 94 consecutive patients who underwent catheter ablation (CA) for symptomatic premature ventricular contractions (PVCs) at OCSC from September 2023 to September 2024. Patients were excluded due to reluctance for participation (*n* = 29), non successful ablation (*n* = 14), and mostly due to the inability to perform a second CPET due to logistical challenges (*n* = 21). Some patients, living far from our center, opted out of follow-up visits as they reported complete symptom resolution post-ablation, deeming further evaluations unnecessary. Final enrollment consisted of 30 patients, all of whom underwent a baseline cardiopulmonary exercise test (CPET) and standardized follow-up.

### 2.2. Pre-Ablation Work-Up

The study included a total of 30 patients with a mean age of 54.93 ± 14.65 years. Among the participants, 19 (63.33%) were men and 11 (36.67%) were women. Patients received standard pre-procedural evaluations, including medical history, 12-lead ECG,24 h Holter monitoring, echocardiography, and CPET. Antiarrhythmic drugs (AADs) were discontinued 48 h prior to CA. PVC burden was quantified, and underlying structural heart disease was excluded. All subjects gave informed consent.

Comorbid conditions were observed in varying frequencies: hypertension and dyslipidemia were present in 12 patients each (40.0%), diabetes in 3 patients (10.0%), smoking and coronary disease in 2 patients each (6.67%), while peripheral artery disease and chronic kidney failure were noted in 1 patient each (3.33%). A history of percutaneous coronary intervention (PCI) was recorded in 1 patient (3.33%), while no patients had a history of coronary artery bypass grafting (CABG).

The mean left ventricular ejection fraction (LVEF) prior to ablation was 54.87 ± 5.86. The mean number of premature ventricular contractions (PVCs) was 23,509.3 ± 10,700.47. Regarding pharmacological therapy, 8 patients were not on any medications, 19 patients were on beta-blockers, and 3 patients were on flecainide. A summary of the above is presented to Table 1.

### 2.3. CPET Protocol Pre Ablation

Cardiopulmonary exercise testing (CPET) was conducted under the Bruce protocol to evaluate the functional capacity and physiological response to exercise in the study population. The mean exercise duration was 9.53 ± 2.03 min, heart rate (Bpm Rest) prior to the test averaged 80.83 ± 16.02 beats per minute, while the maximum heart rate (Bpm Max) achieved during exercise was 145.0 ± 24.07 beats per minute, corresponding to 85% ± 10% of the predicted maximum heart rate. It is worth mentioning that the CPET procedure was terminated due to fatigue in all patients, and no dyspnea or angina were present.

Key CPET parameters indicated preserved aerobic capacity. The mean peak oxygen uptake (VO_2_ max) was 24.97 ± 4.16 mL/kg/min, and the percentage of predicted VO_2_ max was 88% ± 16%, indicating a relatively well-maintained functional reserve. The respiratory exchange ratio (RER) was 1.07 ± 0.13, suggesting maximal effort during exercise testing.

Additional markers of exercise performance included a mean metabolic equivalent (METS) of 7.16 ± 1.17 and an anaerobic threshold (AT) of 15.21 ± 3.25 mL/kg/min, both of which provide insights into the aerobic fitness and metabolic capacity of the population. Ventilation metrics, such as VE max (maximum minute ventilation), were 66.33 ± 16.48 L/min, while the ventilatory equivalents for oxygen (VE/VO_2_) and carbon dioxide (VE/VCO2) were 33.07 ± 6.12 and 31.37 ± 4.79, respectively, suggesting efficient gas exchange dynamics during exercise.

### 2.4. Ablation Procedure

All electrophysiology studies were conducted after an overnight fast and without sedative agents. A three-dimensional navigation platform (CARTO 3 V7; Biosense Webster) guided our activation mapping, using each patient’s PVC ECG signature to pinpoint the arrhythmia source. For PVCs with an LBBB morphology and a V4 transition, we first interrogated the RVOT. If the transition occurred in V3, we extended mapping to include the RVOT, LVOT (covering the aortic cusps), and adjacent coronary venous routes—indirectly targeting the epicardial LV summit via the great cardiac vein. When PVCs showed an LBBB pattern transitioning at V2 or an RBBB pattern, mapping focused on the LVOT, aortic cusps, and coronary vein system. In select cases, a 0.14-inch Visionwire (Biotronik) was advanced into the anterior interventricular vein for unipolar recordings and pace-mapping. A summit epicardial origin was suspected when the earliest activation preceded neighboring sites by the shortest interval at the GCV–proximal AIV junction. An intramural origin at the LV summit was considered possible when the earliest ventricular activation was <15 ms in neighboring anatomical structures [left coronary cusp (LCC), GCV/proximal AIV and basal LV endocardium beneath the GCV course]. Radiofrequency energy delivery (15–40 W, 43 °C) was considered successful if VAs were abolished during ablation and remained absent for at least 30 min after ablation. In the case of an intramural origin, sequential prolonged radiofrequency energy applications at multiple sites displaying similar activation times were performed. The most common site of ablation was the coronary cusps, accounting for 46.15% of cases. The right ventricular outflow tract (RVOT) was the second most frequently targeted site, comprising 26.92% of cases. Ablation within papillary muscles was performed in 11.54% of cases, while 7.69% (Table 2) of ablations were directed at endocardial sites of PVC origin, like septal RV wall or parahisian. Similarly, ablations involving the coronary veins were observed in 7.69% of patients. Patients were evaluated in the outpatient setting at 4 to 8 weeks after ablation and then at 3 months.

### 2.5. CPET Protocol Post Ablation

Following 3 months after ablation, cardiopulmonary exercise testing (CPET) under the Bruce protocol revealed preserved or slightly improved functional capacity across the cohort. The mean exercise duration was 10.0 ± 1.53 min. Heart rate (Bpm Rest) averaged 79.4 ± 19.86 beats per minute, while the maximum heart rate (Bpm Max) reached during exercise was 149.17 ± 26.89 beats per minute, corresponding to 89% ± 12% of the predicted maximum heart rate. Once again CPET procedure was terminated due to fatigue in all patients, and no dyspnea or angina were present.

The mean peak oxygen uptake (VO_2_ max) post-ablation was 26.02 ± 4.34 mL/kg/min, with a percentage of predicted VO_2_ max at 91% ± 16%, indicating a maintained or improved aerobic capacity. The respiratory exchange ratio (RER) was 1.08 ± 0.13, confirming maximal effort during exercise. Metabolic performance metrics included a mean metabolic equivalent (METS) of 7.48 ± 1.24 and an anaerobic threshold (AT) of 16.1 ± 4.07 mL/kg/min, both of which demonstrated slight improvements in the cohort’s exercise efficiency.

Ventilation metrics remained efficient post-ablation, with VE max (maximum minute ventilation) at 69.31 ± 14.72 L/min, ventilatory equivalents for oxygen (VE/VO_2_) at 32.57 ± 4.83, and ventilatory equivalents for carbon dioxide (VE/VCO2) at 31.0 ± 4.57.

### 2.6. Statistical Analysis

All continuous variables were expressed as mean ± standard deviation (SD). Normality of distributions was assessed using the Shapiro–Wilk test. For variables that followed a normal distribution (e.g., VO_2_ max, METS), comparisons between pre- and post-ablation values were made using the paired t-test. For non-normally distributed variables (e.g., NT-proBNP), the Wilcoxon signed-rank test was applied. Categorical variables, such as medication use, were summarized as counts and percentages: no formal statistical testing was applied to these due to the descriptive nature of the analysis.

Effect sizes (Cohen’s d) and 95% confidence intervals (CIs) were calculated for key continuous outcome measures to quantify the magnitude of observed changes. Statistical analyses were conducted using Python 3.11, with functions from the SciPy (v1.11) and statsmodels libraries. Given the exploratory nature and limited sample size of the study, no correction for multiple testing was applied. This has been acknowledged in the Limitations section.

## 3. Results

All ablations were successful, which was a criterion for enrollment, and were verified from the second 24 h Holter. PVCs decreased from 23,509.3 ± 10,700.47 pre-ablation to 1759 ± 1659.15 post-ablation. The mean left ventricular ejection fraction (LVEF) remained stable following ablation, with a pre-ablation value of 54.87 ± 5.86% and a post-ablation value of 54.7 ± 3.63%, showing no statistically significant change.

**Figure 1:**
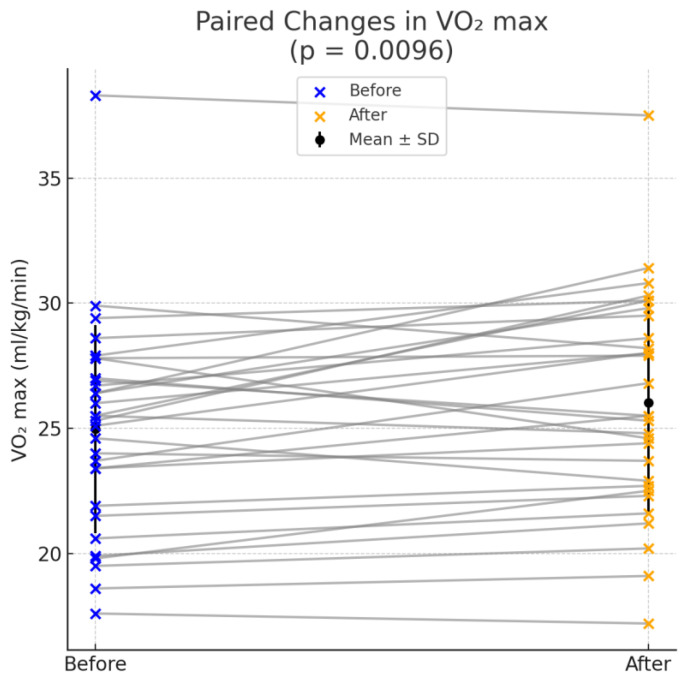
Improvement in peak oxygen uptake (VO_2_ max) following catheter ablation.

**Figure 2:**
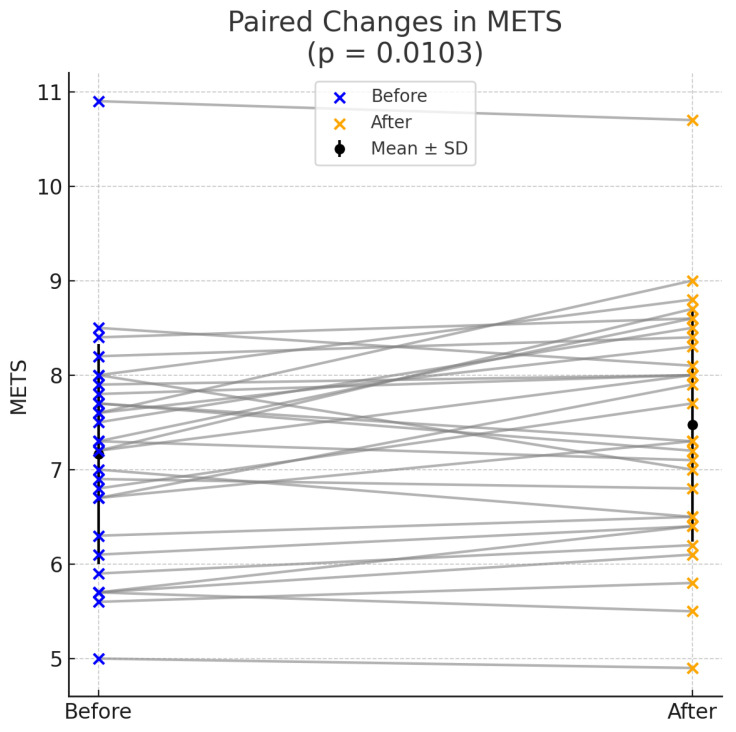
Increase in metabolic equivalent of task (METS) after catheter ablation.

Cardiopulmonary exercise testing (CPET) under the Bruce protocol revealed improvements in two exercise performance parameters following ablation. The mean peak oxygen uptake (VO_2_ max) increased post-ablation (26.02 ± 4.34 mL/kg/min vs. 24.97 ± 4.16 mL/kg/min, *p* = 0.0096), indicating improved aerobic capacity. Similarly, the metabolic equivalent (METS) showed a statistically significant increase (7.48 ± 1.24 vs. 7.16 ± 1.17, *p* = 0.0103). To further evaluate the clinical relevance of the improvements, Cohen’s d effect sizes were calculated. For VO_2_ max, the effect size was 0.506 with a 95% CI of (0.158, 0.903). For METS, Cohen’s d was 0.501 with a 95% CI of (0.144, 0.900). Despite the close mean values on both parameters, before and after ablation, the statistical significance arises from consistent individual improvements across participants, with relatively low variability in the differences better depicted as pair bar plots (left) and box plots (right) who show the overall distribution, median, and spread in Figure 1 and Figure 2. Although there was an observed increase in the percentage of predicted VO_2_ max (91% ± 16% vs. 88% ± 16%), this change did not reach statistical significance (*p* = 0.0793). Other parameters, such as the respiratory exchange ratio (RER) and the anaerobic threshold (AT), demonstrated slight improvements post-ablation but were not statistically significant (RER: *p* = 0.5330, AT: *p* = 0.2368). Similarly, no other parameter, including duration of exercise, changes in heart rate and blood pressure, VE max, VE/VO_2_, or VE/VCO_2_, exhibited a statistically significant change following ablation.

**Figure 3:**
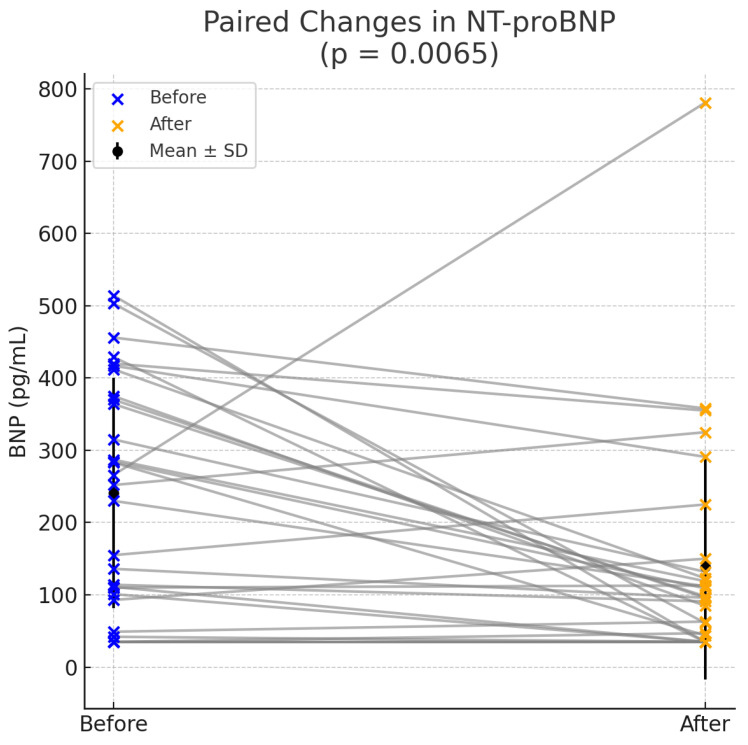
Reduction in NT-proBNP levels after PVC ablation.

At this point it is worth mentioning that after the analysis of Nt-proBNP levels before and after ablation a statistically significant reduction was found. The mean BNP level decreased from 240.93 ± 156.54 pg/mL pre-ablation to 138.47 ± 152.91 pg/mL post-ablation (t = 2.93, *p* = 0.0065), reflecting an improvement in cardiac stress markers. This significant reduction suggests that ablation may contribute to reducing cardiac stress in the study population. Individual patient responses showed variability, with some participants experiencing substantial decreases in BNP levels while others demonstrated minimal changes or slight increases post-ablation (Figure 3).

In terms of medication use, there were notable changes in the pharmacological management of the cohort. Pre-ablation, 26.66% of patients were not on any medication, while 63.33% were on beta-blockers and 10.0% on flecainide. Post-ablation, the proportion of patients not requiring medication increased to 63.33%, while the use of beta-blockers decreased to 36,66%. A summary of the above is presented to Table 3.

## 4. Discussion

Our research demonstrated improvements in VO_2_ max METS which are key parameters reflecting aerobic capacity and exercise tolerance, respectively [8]. These findings underscore enhanced oxygen delivery and utilization during peak exercise. Additionally, a statistically significant reduction in NT-proBNP levels was observed, indicating a decrease in cardiac stress [9] following ablation. In contrast, no significant changes were noted in parameters primarily associated with ventilatory efficiency, such as VE/VO_2_ and VE/VCO_2_ [10]. This differentiation suggests that while PVC ablation improves cardiac efficiency and reduces stress, it does not substantially affect ventilatory mechanisms in this cohort.

VO_2_ max and METS represent the body’s ability to perform and sustain physical activity, making them key indicators of functional capacity and cardiopulmonary health [10]. The improvement in these key parameters after PVC ablation can be explained by the lift of the abnormal sustained pumping efficiency and dyssynchrony of the cardiac muscle under a high burden of PVCs [11].

Premature ventricular contractions (PVCs) disrupt the heart’s normal pumping efficiency by causing intermittent drops in stroke volume and cardiac output. Some PVCs fail to generate enough pressure to open the aortic valve, leading to concealed mechanical bradycardia, which reduces overall cardiac efficiency. This phenomenon not only decreases cardiac output but also increases left ventricular diastolic pressures, resulting in volume overload, which explains the rise in ventricular stress and thus NTproBNP, left ventricular dilation, and systolic dysfunction over time [12]. Furthermore, the lack of a consistent arterial pulse can trigger neuro-vegetative imbalance through baroreceptor reflex activation, further impairing myocardial function [13]. In addition to these mechanical effects, frequent PVCs have been shown to powerfully modulate autonomic control by activating cardiac vagal afferents and altering parasympathetic tone. Animal studies demonstrate that PVCs stimulate both mechanosensitive and chemosensitive neurons in the nodose ganglia more strongly than ischemia, leading to impaired baroreflex sensitivity and reduced high-frequency heart rate variability—hallmarks of parasympathetic withdrawal. Although we did not directly measure autonomic parameters in this cohort, the improvements in CPET performance post-ablation may partly reflect restoration of neuro-vegetative balance once ectopic activity is eliminated. By mitigating aberrant afferent signaling, catheter ablation could normalize baroreflex function and enhance parasympathetic tone, contributing to both symptom relief and objective gains in exercise capacity [14].

Catheter ablation effectively reduces PVC burden, restoring a more regular heart rhythm. This improves stroke volume and cardiac output, allowing for better oxygen delivery to tissues [2]. Matteucci et al. recently reported that successful atrial fibrillation ablation markedly improves patient-reported quality-of-life and symptom burden [15]. These findings reinforce the principle that restoration of a regular cardiac rhythm confers significant functional benefits. Importantly, the observed improvements in VO_2_ max following PVC ablation may reflect enhanced peripheral oxygen extraction by skeletal muscles. By alleviating the intermittent perfusion deficits caused by PVCs, ablation helps make oxygen delivery more efficient during exercise. Supporting this, studies have shown that interventions that improve oxygen delivery and peripheral extraction, such as exercise training or the correction of arrhythmias, result in significant VO_2_ max improvements, even without altering ventilatory efficiency or respiratory exchange ratio (RER) parameters [16].

While this study demonstrates improvements in functional capacity and NT-proBNP following PVC ablation, the small sample size restricted our ability to conduct a meaningful multivariate analysis to identify clinical predictors of improvement. Preliminary observations suggest potential trends—for example, patients with outflow tract PVCs and those with baseline lower VO2 max appeared to benefit more significantly, although this did not reach statistical significance. Moreover, although a range of PVC origins were included—including RVOT, LVOT, coronary cusps, papillary muscles, and epicardial sites—we noted that patients with outflow tract PVCs tended to show greater improvements in VO_2_ max and NT-proBNP compared to those with PVCs arising from papillary muscles or epicardial locations. While these trends were not statistically significant, they align with previous studies highlighting better outcomes and procedural profiles in outflow tract PVCs [17]. Similarly, sex-related differences and the site of ablation may influence outcomes, but larger studies are required to explore these factors systematically.

In addition to the improvements in functional and biomarker outcomes, our study also revealed a significant reduction in beta-blocker use following ablation—from 63.3% of patients receiving beta-blockers pre-ablation to only 36.7% post-ablation. This transition is particularly important for older or polymorbid individuals, in whom beta-blockers may be poorly tolerated due to fatigue, bradycardia, or hypotension [18]. Reducing medication burden not only lessens side effects but may also enhance quality-of-life and adherence to long-term management.

### Limitations

This study has several limitations. First, the small sample size (N = 30) limits the generalizability of the findings to larger populations. Second, the study was conducted at a single-center, which may introduce selection bias and reduce external validity. Third, patients with unsuccessful ablation or impaired cardiac function were excluded, something that is a loss since the patients of that profile may benefit the most from CA. Additionally, the lack of long-term follow-up data prevents an assessment of sustained improvements in functional capacity and cardiac stress markers, rather than only those in a 3 month timespan. It is also important to acknowledge that post-procedural changes in medication, such as the discontinuation of beta-blockers in some patients, could have contributed to the observed improvements in functional capacity, inde-pendent of the ablation Lastly, while NT-proBNP reductions were significant, variabil-ity among patients warrants further investigation to identify factors influencing these results. Furthermore, although normality testing was performed and appropriate sta-tistical methods were applied (e.g., Wilcoxon signed-rank test for non-normal data), the small sample size remains a limiting factor in interpreting these results and no formal correction for multiple comparisons was applied, given the exploratory nature of this study.

## 5. Conclusions

The observed improvements in VO_2_ max, METS, and NTproBNP following PVC ablation are primarily due to the reduction in arrhythmic burden, which enhances cardiac efficiency and alleviates cardiac stress during exercise. Other parameters, such as LVEF and certain ventilatory metrics, may remain unchanged post-ablation, especially in patients without significant structural heart disease. These findings underscore the specific benefits of CA in improving functional exercise capacity through rhythm normalization.

## Figures and Tables

**Table 1:** Baseline Characteristics.

Variable	Value
Age (years)	54.93 ± 14.65
Men	19 (63.33%)
Women	11 (36.67%)
Hypertension	12 (40.0%)
Dyslipidemia	12 (40.0%)
Diabetes	3 (10.0%)
Smoking	2 (6.67%)
Coronary disease	2 (6.67%)
History of PCI	1 (3.33%)
History of GABG	0 (0.0%)
Peripheral artery disease	1 (3.33%)
Chronic kidney failure	1 (3.33%)
Medication before ablation	None: 8 (26.66%), beta-blocker: 19 (63.33%), and flecainide: 3 (10.0%)

**Title:** Demographic and clinical characteristics of the study population. **Legend:** This table summarizes baseline demographics and comorbidities for the 30 patients who underwent successful PVC ablation.

**Table 2:** Procedural Characteristics.

Variable	Value
Ablation site	
Right ventricular outflow tract (RVOT)	26.92%
Coronary cusps	46.15%
Papillary muscles	11.54%
Endocardial sites (RV septal, parahisian)	7.69%
Coronary veins	7.69%

**Title:** Ablation site distribution. **Legend:** Distribution of ablation sites among patients. Detailed procedural time, radiofrequency time, and fluoroscopic time were not included in the final dataset and are therefore not available in this analysis.

**Table 3:** Outcome measures before and after ablation.

Variable	Before Ablation (Mean ± SD)	After Ablation (Mean ± SD)	*p*-Value
PVC burden	23,509.3 ± 10,700.47	1759 ± 1659.15	<0.001
LVEF	54.87 ± 5.86	54.7 ± 3.63	NS
NT-proBNP	240.93 ± 156.54	138.47 ± 152.91	0.0065
VO_2_ max	24.97 ± 4.16	26.02 ± 4.34	0.0096
METS	7.16 ± 1.17	7.48 ± 1.24	0.0103
% Predicted VO_2_	0.88 ± 0.16	0.91 ± 0.16	0.0793
RER	1.07 ± 0.13	1.08 ± 0.13	0.5330
AT	15.21 ± 3.25	16.1 ± 4.07	0.2368
VE max (l/min)	66.33 ± 16.48	69.31 ± 14.72	NS
VE/VO_2_	33.07 ± 6.12	32.57 ± 4.83	NS
VE/VCO_2_	31.37 ± 4.79	31.0 ± 4.57	NS
Resting heart rate	80.83 ± 16.02	79.4 ± 19.86	NS
Max heart rate	145.0 ± 24.07	149.17 ± 26.89	NS
% Max HR predicted	0.85 ± 0.10	0.89 ± 0.12	NS
Exercise duration	9.53 ± 2.03	10.0 ± 1.53	NS
Medication after ablation	None: 8 (26.66%), beta-blocker: 19 (63.33%), and flecainide: 3 (10.0%)	None: 18 (63.3%), beta-blocker: 12 (36.6%), and flecainide: 0 (0.0%)	↑ None, ↓ beta-blocker, and flecainide discontinued

**Title:** Changes in electrophysiological, functional, and biomarker parameters Following PVC ablation. **Legend:** This table compares outcome measures before and after ablation. Values are expressed as mean ± SD. NS = not statistically significant.

## Data Availability

The data presented in this study are available on request from the corresponding author. The data are not publicly available due to privacy and ethical restrictions.

## Abbreviations

PVCs	Premature Ventricular Contractions
CA	Catheter Ablation
CPET	Cardiopulmonary Exercise Testing
LV	Left Ventricle
NT-proBNP	N-terminal pro B-type Natriuretic Peptide
LVEF	Left Ventricular Ejection Fraction
METS	Metabolic Equivalents
AT	Anaerobic Threshold
VE/VO_2_	Ventilatory Equivalent for Oxygen
VE/VCO_2_	Ventilatory Equivalent for Carbon Dioxide
RER	Respiratory Exchange Ratio
GCV	Great Cardiac Vein
LVOT	Left Ventricular Outflow Tract
RVOT	Right Ventricular Outflow Tract
AIV	Interventricular Vein
SOO	Site of Origin
LBBB	Left Bundle Branch Block
RBBB	Right Bundle Branch Block
VA	Ventricular Arrhythmia
AADs	Antiarrhythmic Drugs
PCI	Percutaneous Coronary Intervention
CABG	Coronary Artery Bypass Grafting
OCSC	Onassis Cardiac Surgery Center
